# A Walk-In Screening of Dementia in the General Population in Taiwan

**DOI:** 10.1155/2014/243738

**Published:** 2014-05-04

**Authors:** Chun-Hung Chen, Ling-Chun Wang, Tzu-Chiao Ma, Yuan-Han Yang

**Affiliations:** ^1^Department of Neurology, Kaohsiung Medical University Hospital, Kaohsiung Medical University, Kaohsiung City 80708, Taiwan; ^2^Mentality Protection Center, Fo Guang Shan Compassion Foundation, Kaohsiung City 80050, Taiwan; ^3^Graduate Institute of Oral Health Sciences, Kaohsiung Medical University, Kaohsiung City 80708, Taiwan; ^4^Department of Neurology and Master's Program in Neurology, Faculty of Medicine, Kaohsiung Medical University, Kaohsiung City 80708, Taiwan; ^5^Department of Neurology, Kaohsiung Municipal Ta-Tung Hospital, Kaohsiung Medical University, Kaohsiung City 80145, Taiwan

## Abstract

Alzheimer's disease (AD) has increased in its prevalence due to the increasing aged population. Currently there is no updated data on the prevalence of dementia including its very mild stage in Taiwan. Under the extensive coverage of Mentality Protection Center (MPC), Fo Guang Shan, Taiwan, the volunteers of MPC have conducted the medicine-related services and the screening of dementia by AD8 (ascertainment of dementia 8) that can screen the dementia even at its very mild stage in general population in all Taiwan. From 2011 to 2013, in total, 2,171 participants, 368 in the northern, 549 in the central, 877 in the southern, and 377 in the eastern part, were recruited with the mean age being 66.9 ± 10.2 years old. The ratio of suspected dementia patients, AD8 score greater than or equal to 2, was 13.6% of all recruited participants with their mean AD8 score being 2.9 ± 1.3, mean age being 69.4 ± 10.8 years old, and female predominance being 73.0%. Although this is a screening study, it has extensive coverage of all Taiwan and the use of AD8 is capable of screening very mild dementia. A further study with a randomized sampling to examine the prevalence and incidence of dementia including its very mild stage is encouraged.

## 1. Introduction

Alzheimer's disease (AD) is the main cause of dementia in Taiwan [1]. At present, most available data and studies regarding the prevalence of AD in Taiwan were completed in the past two decades [2–7], and these studies were limited to certain areas, Kaohsiung city, Kinmen county, Taipei city, or Ilan city, which do not provide an overall understanding of dementia in Taiwan [1]. Moreover, the aged population has been increasing steadily in recent years. These published studies did not have sufficient data to reflect current status of the aged population. These published studies also did not sufficiently detect dementia at the very mild stage, and did not address the very mild stage of dementia. To date, little is known about the current status of dementia in the general population in Taiwan.

AD8 is a brief tool used to screen dementia, which was developed from Washington University in St. Louis [8], which is capable of screening very mild dementia in a general population and is used extensively in other countries, including Taiwan [9]. If the screening result of AD8 is 2, the individual would be considered having dementia including very mild dementia [9]. AD8 can be administered to an informant of demented patients and the patient himself with a similar discriminative rate to help with differentiating demented from nondemented subjects [10].

The Mentality Protection Center (MPC) is a nonprofit institution and was established under Compassion Foundation Fo Guang Shan in 2008 to provide medical and charitable services to the general population in the world through hundreds of branches of Fo Guang Shan. Working together with the branches of Fo Guang Shan, scattered throughout urban, suburban, and rural areas in each of the northern, central, southern, and eastern parts of Taiwan, the MPC has launched dementia-screening projects to assist the elderly population regarding screening for dementia by utilizing AD8 from 2011 to 2013. The screening project, including dementia, depression, and sleep disorder, was administrated to the voluntary walk-in aged population in these branches if they were aware of the medical service and the dementia-screening project. All of the screening results were recruited to the MPC headquarter for the statistical analyses.

## 2. Material and Methods

### 2.1. The Training of Interviewers

All of our interviewers were senior nurses or other medical-related specialists. Before their administration of AD8 to the elderly population, they had to receive a series of training courses with regard to dementia-related and medical-related topics and to practice the administration of AD8 with the general population by working with experienced interviewers and physicians with internships. All of the interviewers were volunteers of MPC and completed walk-in screenings in this project in Taiwan.

### 2.2. Walk-In Screening

There are 59 branches of the Mentality Protection Center in Taiwan. All of these branches are scattered throughout the northern, central, southern, and eastern parts of Taiwan and distributed in urban, suburban, and rural areas in each part of Taiwan. From March 1, 2010 to April 30, 2013, 53 walk-in screenings were conducted at these 59 branches of MPC. Every screening in a branch had to last for 1 day to provide the medical and charitable service to the general elderly population and the screening of dementia by AD8 [9], depression by Center for Epidemiologic Studies-Depression (CES-D) scale [11], and sleep disorder by Pittsburgh Sleep Quality Index (PSQI) [12]. For all 53 screenings, 7 screenings were conducted in the northern part, 19 in the central part, 24 in the southern part, and 3 in the eastern part of Taiwan to the general population if they were older than 50 years old. Every walk-in participant was voluntary to join and complete all screens.

### 2.3. Participants and Evaluations

All participants voluntarily joined the screening activity without any reward. CES-D, AD8, and PSQI were administrated to individuals after identifying age, gender, and living area. The individual was considered as suspected dementia if his/her AD8 total score was greater than 2. Using these existed data for the statistical analyses was approved by the Kaohsiung Medical University Hospital Institutional Review Board (IRB). All the information related to the privacy or that can be identified was not recorded during the screening process.

### 2.4. Statistics

Data analysis was performed using SPSS (version 12.0.1 for Windows, SPSS Inc., Chicago, IL, USA). All statistical tests were two-tailed and an alpha of 0.05 was taken to indicate significance. Analysis of variance (ANOVA) was used to compare the difference of group mean for age and for AD8 total score among the four areas of Taiwan for all participants and for all suspected dementia individuals. *t*-test was used to compare the group mean of age and of AD8 total score between suspected dementia and nondemented individuals. Chi-square test was used to compare the proportion of each reported subitem of AD8 and gender between suspected dementia and nondementia subjects and among four areas, the northern, central, southern, and eastern parts of Taiwan.

## 3. Results

In total, 2,171 participants, 368 in the northern, 549 in the central, 877 in the southern, and 377 in the eastern part, were recruited with the mean age 66.9 ± 10.2 years old. The mean age was significantly different among four areas (*P* < 0.001). The age of participants from the eastern area were older, 70.0 ± 10.0 years old, than the other three areas (Table 1). Participants were predominantly female, and there were no significant differences with regard to gender proportion (*P* = 0.485) although it was higher in the southern area (71.5%), compared to others (Table 1). For the proportion of reported change in each subitem of AD8, there was a significant difference in AD8-2 (reduced interest in hobbies and other activities) among the 4 areas (*P* = 0.024), where the eastern area had a higher proportion (10.9%). Similarly, there was a significant difference in AD8-3 (repeating of questions, stories, or statements) (*P* < 0.001) among the 4 areas, where the northern area had a higher proportion (13.6%). In the general population, the top two frequently reported items were AD8-8 (consistent problems with thinking and/or memory) (13.3%), followed by AD8-5 (forgetting correct month or year) (8.2%) (Table 1).

The mean age between suspected demented (69.4 ± 10.8) and nondemented subjects (66.6 ± 10.0) was significantly different (*P* < 0.001). The ratio of suspected demented subjects was not significantly different among the four areas (*P* = 0.854) as well as age (*P* = 0.162), gender (0.383), and mean AD8 total score (*P* = 0.658) (Table 2). In general, the ratio of suspected dementia patients was 13.6% of all recruited participants with their mean AD8 score being 2.9 ± 1.3, mean age being 69.4 ± 10.8 years old, and female predominance being 73.0% (Table 2).

Ratio of reported change for each subitem of AD8 and AD8 total score were significantly different between suspected demented and nondemented group (*P* < 0.001 for each comparison of AD8 subitem and AD8 total score) (Table 3). In nondemented group, the AD8-8 (consistent problems with thinking and/or memory (6.4%)), AD8-2 (reduced interest in hobbies/activities were frequently reported (3.1%)), and AD8-5 (forgetting correct month or year (3.0%)) were the top 3 frequently reported change in AD8 subitems (Table 3). Alternatively, these proportions of AD8 subitems in nondemented subjects, in part, were similar to those in suspected demented subjects where the AD8-8 (consistent problems with thinking and/or memory (56.8%)), AD8-7 (difficulty in remembering appointments (47.0%)), and AD8-5 (forgetting correct month or year (40.9%)) were frequently reported (Table 3).

## 4. Discussion

This study provides updated information on the current status of dementia and the frequently reported presentations for mild dementia of Taiwan. Although the study design did not utilize a randomized sampling method to examine the prevalence and incidence of dementia in Taiwan, it provided information of dementia coming from four areas, the northern, central, southern, and eastern parts of Taiwan, for an overall examination for dementia and reported the frequently reported complaints and symptoms of dementia, including very mild dementia.

Prevalence studies of dementia in Taiwan were mostly conducted 2 decades ago [2–7], which cannot reflect our current status. Importantly, those studies were also done sporadically and separately in a localized area of Taiwan with different study designs and various psychometrics so it was not easy to have this data put together to reflect the condition of dementia for all of Taiwan. Meanwhile, some of those studies were not capable of reflecting the status of very mild dementia, CDR0.5 [13], that was less reported and addressed in those studies [2–7].

Most of our recruited participants were female predominant with a mean age of 66.9 ± 10.2 years old. Such results might, in part, be related to a female predominance for people aged greater than 50 years old in Taiwan [14], and females might be more inclined to participate in this social facility as compared with male, especially with regard to religion.

For the significant discrepancies in reported change of the AD8 subitem, the proportion of AD8-3 (repeats questions, stories, or statements) was higher in the northern part (13.6%), where participants mostly come from the greater Taipei area, the greatest population density, [15] that is the location of the capital and having a higher daily cost of living caused the residents to be more anxious and stressed. The anxiety and stress will cause people to have memory problems and to repeat questions, stories, or statements [16, 17]. AD8-2 (reduced interest in hobbies/activities) was found to be higher (10.9%) in the eastern part of Taiwan, as compared to others. The participants recruited from eastern part mainly came from Taitung City and Taitung County which have the lowest population density [15] and less flourishing and prosperous economic status as compared to other areas in Taiwan. As such, the residents in those areas have few interests and hobbies and have quite unsatisfactory levels of living status, that may have influence the higher proportion of reported AD8-2.

These suspected demented patients, AD8 ≥ 2, were also female predominant, which was similar to our previous publication that current demented patients were female predominant [18], and also in other publications that female were predominant in the prevalence of AD [19]. The mean age of these suspected demented subjects was younger than that of our previously reported mean age, 79.3 ± 7.7, for current AD patients in Taiwan [18]. The difference was mainly due to the recruited subjects in our previous study that were from a hospital-based study where a more advanced stage of demented patients are treated, especially in sites such as medical centers.

In the reported AD8 subitems, although each subitem of AD8 was shown to have a significant difference between suspected demented and nondemented subjects, there were differences in the top three reported AD8 subitems between them. The different items were the second high frequency of reported change of subitems, AD8-7 in suspected dementia and AD8-2 in nondementia subjects. AD8-7 (difficulty remembering appointments) was mainly from the result of a memory problem in which an individual cannot memorize recent events, the impairment of recent memory could be the initial presentation of very mild dementia, especially in AD [20, 21].

Individuals with depressive syndrome tended to lose interest and to have the memory problems [19, 22]. The prevalence of depressive syndrome among community-dwelling elderly in Taiwan was 27.5% [23] and that would result in our nondemented subjects having reports of AD8-8, AD8-2, and AD8-5 subitems, with reduced interest and having memory problems.

Our study has several strengths worth highlighting. First, we used the same interviewers to administer the AD8 instrument in all screenings so that we would not have interrater differences with regard to the study results. Second we used the same instrument, AD8, to avoid the biases of different tools used in different screening sites, and since AD8 is capable of screening very mild dementia [8–10], it would be more sensitive as compared to other tools used in the past such as MMSE [2–4]. Third, we surveyed individuals in the northern, central, southern, and eastern parts of Taiwan to reflect the status of screening results for all of Taiwan, which should be more objective when compared to other published studies in limited areas [2–7] in order to more accurately reflect and report on the status of dementia in Taiwan. On the other hand, this study involves a number of limitations that need to be addressed. Firstly, this study did not involve randomized sampling of the general population and as a result our study cannot reflect the real prevalence and incidence of current dementia in Taiwan. Secondly, we only used AD8 without other laboratory data or imaging data to provide the clinical diagnosis of each suspected demented patient; however, AD8 has been validated as being reliable in terms of sensitivity and specificity in screening dementia [9] and this study focused on the screening, not the diagnosing of dementia. Third, the study was a walk-in screening. This means that only the individuals who could independently walk into or be brought into our branches for screening were recruited. If he/she was independent or could not be brought into our screening sites, he/she might not join the screening process so that our current study may therefore underestimate the real status of the prevalence of dementia.

This was a screening study performed with some limitations and strengths, but did involve extensive coverage of all Taiwan and the use of AD8 was capable of screening very mild dementia. A further study with a randomized sampling to examine the prevalence and incidence of dementia with its subtypes in Taiwan is encouraged.

## Figures and Tables

**Table 1:** Demographic characteristics of all recruited participants.

	Northern	Central	Southern	Eastern	*P* value	Total
Number (*n*, %)	368 17.0%	549 25.3%	877 40.4%	377 17.4%		2171 100%
Age, years (mean ± SD)	68.7 ± 9.8	65.0 ± 10.0	66.2 ± 10.1	70.0 ± 10.0	<0.001	66.9 ± 10.2
Female (*n*/*N*, %)	258 70.1%	383 69.8%	627 71.5%	253 67.1%	0.485	1521 70.1%
AD8 score (mean ± SD)	0.6 ± 1.0	0.6 ± 1.1	0.6 ± 1.1	0.5 ± 1.1	0.715	0.6 ± 1.1
AD8-1*	15, 4.1%	13, 2.4%	27, 3.1%	6, 1.6%	0.184	61, 2.8%
AD8-2*	18, 4.9%	41, 7.5%	68, 7.8%	41, 10.9%	0.024	168, 7.7%
AD8-3*	50, 13.6%	39, 7.1%	52, 5.9%	27, 7.2%	<0.001	168, 7.7%
AD8-4*	8, 9.6%	26, 4.7%	39, 4.4%	10, 2.7%	0.098	83, 3.8%
AD8-5*	25, 6.8%	52, 9.5%	76, 8.7%	25, 6.6%	0.304	178, 8.2%
AD8-6*	16, 4.3%	26, 4.7%	53, 6.0%	19, 5.0%	0.565	114, 5.3%
AD8-7*	29, 7.9%	38, 6.9%	81, 9.2%	24, 6.4%	0.253	172, 7.9%
AD8-8*	45, 12.2%	71, 12.9%	127, 14.5%	45, 11.9%	0.551	288, 13.3%

*Change in each AD8 subitem (number and percentage in that area).

AD8-1: problems with judgment; AD8-2: reduced interest in hobbies/activities; AD8-3: repeats questions, stories, or statements; AD8-4: trouble learning how to use a tool, appliance, or gadget; AD8-5: forgetting correct month or year; AD8-6: difficulty handling complicated financial affairs; AD8-7: difficulty remembering appointments; AD8-8: consistent problems with thinking and/or memory.

**Table 2:** Demographic characteristic of participants suspected dementia*.

	Northern (*N* = 368)	Central (*N* = 549)	Southern (*N* = 877)	Eastern (*N* = 377)	*P* value	Total (*N* = 2171)
*n*, %	48, 13.0%	74, 13.5%	126, 14.4%	48, 12.7%	0.854	296, 13.6%
Age, mean ± SD	70.4 ± 9.9	67.7 ± 11.9	68.9 ± 9.5	71.9 ± 12.5	0.162	69.4 ± 10.8
Gender (Female/*n*, %)	36, 75.0%	55, 74.3%	98, 77.8%	27, 56.3%	0.383	216, 73.0%
Mean AD8 score	2.7 ± 0.9	3.0 ± 1.3	2.8 ± 1.2	2.9 ± 1.6	0.658	2.9 ± 1.3

*Defined as AD8 total score ≧ 2.

**Table 3:** AD8 subitems in dementia and nondementia subjects.

AD8 subitems	Dementia (*N* = 296)	Nondementia (*N* = 1875)	*P* value
Age	69.4 ± 10.8	66.6 ± 10.0	<0.001
AD8-1 (*n*, %)	40, 13.5%	21, 1.1%	<0.001
AD8-2	109, 36.8%	59, 3.1%	<0.001
AD8-3	114, 38.5%	54, 2.9%	<0.001
AD8-4	66, 22.3%	17, 0.9%	<0.001
AD8-5	121, 40.9%	57, 3.0%	<0.001
AD8-6	86, 29.1%	28, 1.5%	<0.001
AD8-7	139, 47.0%	33, 1.8%	<0.001
AD8-8	168, 56.8%	120, 6.4%	<0.001
Total score	2.9 ± 1.3	0.2 ± 0.4	<0.001

AD8-1: problems with judgment; AD8-2: reduced interest in hobbies/activities; AD8-3: repeats questions, stories, or statements; AD8-4: trouble learning how to use a tool, appliance, or gadget; AD8-5: forgetting correct month or year; AD8-6: difficulty handling complicated financial affairs; AD8-7: difficulty remembering appointments; AD8-8: consistent problems with thinking and/or memory.
